# Interventional Radiology Suite: A Primer for Trainees

**DOI:** 10.3390/jcm8091347

**Published:** 2019-08-30

**Authors:** Bedros Taslakian, Ross Ingber, Eric Aaltonen, Jeremy Horn, Ryan Hickey

**Affiliations:** 1 Vascular and Interventional Radiology Section, Department of Radiology, NYU Langone Health, New York, NY 10016, USA; 2 Northwell Health, Manhasset, NY 11030, USA

**Keywords:** interventional radiology, trainees

## Abstract

Familiarity with different instruments and understanding the basics of image guidance techniques are essential for interventional radiology trainees. However, there are no structured references in the literature, and trainees are left to “pick it up as they go”. Puncture needles, guidewires, sheath systems, and catheters represent some of the most commonly used daily instruments by interventional radiologists. There is a large variety of instruments, and understanding the properties of each tool will allow trainees to better assess which type is needed for each specific procedure. Along with understanding the tools required to perform various interventional radiology procedures, it is important for trainees to learn how to organize the room, procedural table, and various equipment that is used during the procedure. Minimizing clutter and improving organization leads to improved efficiency and decreased errors. In addition, having a fundamental knowledge of fluoroscopy, the most commonly used imaging modality, is an integral part of beginning training in interventional radiology.

## 1. Introduction

Interventional radiology (IR) is an expanding specialty requiring years of training to master the skills needed to perform the wide breadth of minimally invasive procedures. The first step in this training is to become familiar with the basic equipment found within every IR suite and learn how to properly prepare for each procedure. It is important for trainees to know how to organize an IR suite before beginning a procedure, as well as techniques for optimizing image quality while being savvy about radiation protection. Once this information is known, trainees can continue to learn the clinical indications for and technical aspects of each IR procedure.

Although many interventional radiologists have preferential wires, sheaths, and catheters for performing procedures, it is imperative that trainees know the wide variety of tools available in the IR armamentarium. While newly shaped and designed catheters, wires, and other equipment continue to be created for select applications, an understanding of the basic properties of commonly used instruments is essential for trainees, as well as practicing interventional radiologists. 

Proper organization and preparation is fundamental to any procedural specialty. While many aspects of IR are similar to that of surgery, most often, interventional radiologists are themselves required to set up equipment and organize the procedural table. Preprocedural planning and intraprocedural organization is important for time management, and more importantly, for patient safety. Planning errors, as well as intraprocedural equipment unavailability have been shown to lead to errors during IR procedures [1]. 

Fluoroscopy, ultrasound, and computed tomography are necessary for a wide range of procedures performed by interventional radiologists. There are several variables involved in acquiring fluoroscopic images to ensure the best image quality while protecting both the patient and operator from excess radiation [2]. Although information regarding equipment, room organization, and fluoroscopic imaging techniques are expected to be learned by all interventional radiologists, there are no structured sources to provide this information. This article seeks to provide a reference for basic components of IR procedures that must be understood by trainees and practicing interventional radiologists for procedural success.

## 2. Basic Interventional Tray

### 2.1. Puncture Needles

**Figure 1:**
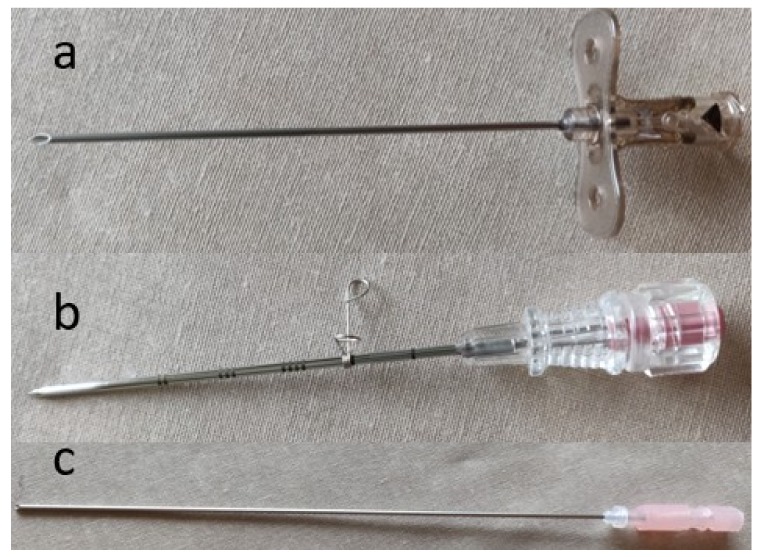
Photograph shows basic needles. (a) Single-wall, hollow-core needle, (b) Trocar needle, and (c) Chiba needle.

Regardless of the procedure, the initial step in every IR procedure is access. Various access needles are available depending on the intended procedure. Needles may be simple, one-piece, bevel-tipped access needles, or a coaxial system in which there is a stylet within an outer metal cannula. A single-wall cutting needle has a hollow core and a beveled (angled) tip that allows sturdy access to vessels and can cut through fibrotic tissue (e.g., post-operative groin) (Figure 1a). The needle often has a small notch on the hub that corresponds to the orientation of the bevel. A trocar needle is an example of a coaxial system in which there is either a beveled or non-beveled outer cannula that contains an inner removable sharp three-sided needle (Figure 1b). This type of needles is less steerable than a cutting needle; however, it allows the operator to leave in place the blunt cannula within the targeted vessel or organ for exchanges. A Chiba needle is another example of a coaxial system in which the outer cannula and inner needle are both beveled (Figure 1c). This beveling allows the needle to be better steered, as compared to a trocar needle, and is often used for biliary and renal access. Beveled needles are more steerable, because they bend away from the bevel.

**Figure 2:**
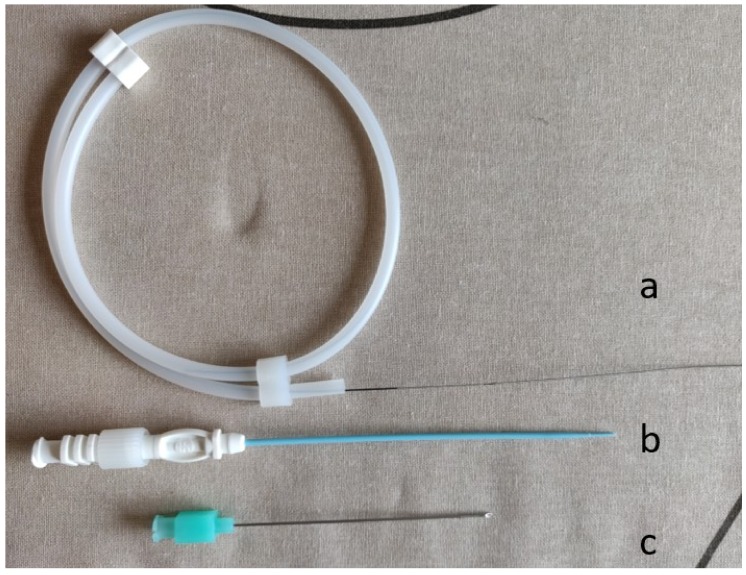
Micropunture system. (a) Microwire (0.018-inch wire), (b) coaxial dilator, and (c) micropuncture needle (21 guage).

Needle diameter is measured in gauge, which always indicates the outer diameter of the needle. The lower the gauge, the larger the needle [3]. It is essential to know the inner diameter of the needle used, as this determines the size of wire that can be advanced through the needle. Although the inner diameter of the needle varies depending on the thickness of the needle wall, in general, an 0.018-inch wire will pass through most 22-gauge or larger needles. An 0.035-inch wire generally requires a 19-gauge or larger needle. Micropuncture systems are often used for difficult access situations (e.g., small target, thrombolysis, antegrade femoral puncture) to decrease the rate of access site complications (Figure 2). The initial puncture is relatively error-tolerant, because it is performed with a 21-gauge needle that allows for the insertion of an 0.018-inch access guidewire. Then, the needle is exchanged for a coaxial dilator, allowing conversion to a larger sheath or catheter over a 0.035-inch wire. 

### 2.2. Guidewires

Guidewires are often used to facilitate guidance through structures (i.e., vessels, biliary/urinary system), or to exchange various devices. There are numerous types of wires with varying properties to be considered for each procedure. Wire properties include diameter (measured in inches, outer diameter), stiffness, length, and hydrophilicity. Wires generally come in a range of sizes from 0.010–0.038 inches, although 0.014-inch, 0.018-inch, and 0.035-inch wires are the most common. The wire should be tightly matched to the needle’s and/or catheter’s end hole. If the guidewire is too big compared to the end hole of the needle or catheter, it will jam. Advancing a device over a guidewire much smaller than the end hole of the device may cause vessel injury or prevent smooth movement over the guidewire.

**Figure 3:**
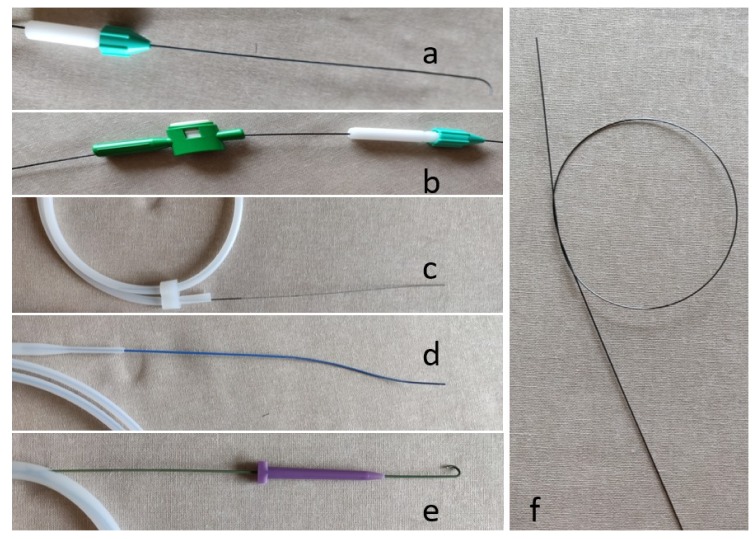
Different types of wires and torque devices. (a) Hydrophilic wire with a mounted torque device, (b) two different torque devices mounted on a hydrophilic wire, (c) access wire with a floppy tip, (d) Amplatz wire, (e) J-shaped Rosen wire, and (f) access wire with a coiled end to facilitate torquing the wire without the need for a torque device.

Hydrophilic wires are less thrombogenic compared to non-hydrophilic wires, and are useful in fluid environments when less resistance is desired [4] (Figure 3a). These wires are slippery when wet and sticky when dry. When using hydrophilic wires, it is important to keep the wire wet to activate the hydrophilic properties of the wire. Additionally, the use of a torque device (Figure 3a,b) allows the operator to better manipulate hydrophilic wires, and can assist in pinning the hydrophilic wire as the catheter is advanced. Although some wires are not technically hydrophilic (e.g., Bentson wire), wiping these wires down with a gauze soaked in heparinized saline often helps facilitate exchanges with various catheters and tools, and helps prevent thrombus formation around the wire, which can be stripped and dislodged during wire exchanges. 

There are three main types of guidewires: access wires, maneuver wires, and rail wires (Table 1). After obtaining access with a needle, an access wire is advanced into the targeted vessel or structure. Once the access wire is in place, the needle can be exchanged for a sheath, dilator, or catheter. Access wires are often short and have a floppy tip in order to be as atraumatic to internal structures as possible (Figure 3c). As the name implies, maneuver wires are used to navigate throughout the vasculature and sub-select target vessels or structures. There are many different maneuver wires that have varying properties to assist with manipulation and achieve the desired outcome, including various sizes, tip shapes, and lengths of the floppy leading end.

Rail wires are stiffer wires that are ideal for equipment exchanges. The most commonly used rail wires are the Amplatz (Boston Scientific, Marlborough, MA) (Figure 3d) and Rosen (Cook Medical, Bloomington, IN) (Figure 3e) wires. These wires come in varying sizes, including exchange length wires for longer devices. An Amplatz wire has a floppy tip and stiffer body, and is used when advancing heavier/larger devices, performing angioplasty, or deploying a stent. The Rosen wire is a J-shaped wire that has an intermediate stiffness all the way throughout the wire (Figure 3e). The J-shaped tip is ideal for intravascular procedures because it helps prevent any intraluminal trauma: the leading J edge is blunt, and helps avoid branch vessels. Stiff wires should not be steered through curves as it can cause injury; instead, they should always be introduced through a catheter. When using non-hydrophilic wires with an angled tip, coiling the end of the wire at the operator’s end helps direct the wire without the use of a torque device (Figure 3f).

There are several important pearls that operators should know regarding wire exchanges and manipulations. When exchanging equipment over a wire, it is essential to always maintain control of the wire (e.g., the back end of the wire must be completely through the device before advancing the device within the patient). This ensures that the wire is not accidently pushed within the patient when advancing the device over the wire. To maintain the guidewire position, the length of the exchange wire must be longer than the sum of the catheter or device length and the distance from the puncture site to the target structure [3]. For simplicity, when choosing an exchange length wire, the wire should be at least twice the length of the catheter or device that is being exchanged. When performing exchanges over wires with floppy tips, the operator must ensure that the stiff part of the wire is within the targeted area, as trying to exchange over only the floppy portion could result in loss of access. It is essential to avoid the use of hydrophilic wires for initial access because of the difficulty of stabilizing them during exchanges and concerns of shearing and subsequent embolization of the wire coating when the wire is manipulated through the puncture needle. In addition, when possible, over-the-wire exchanges, angioplasty, or stenting should not be performed over hydrophilic wires. Finally, the back end of the wire should never be advanced through a catheter into a vessel, as it lacks the floppy tip of the front end, and could cause traumatic dissection or even the perforation of a vessel. 

### 2.3. Sheath Systems

**Figure 4:**
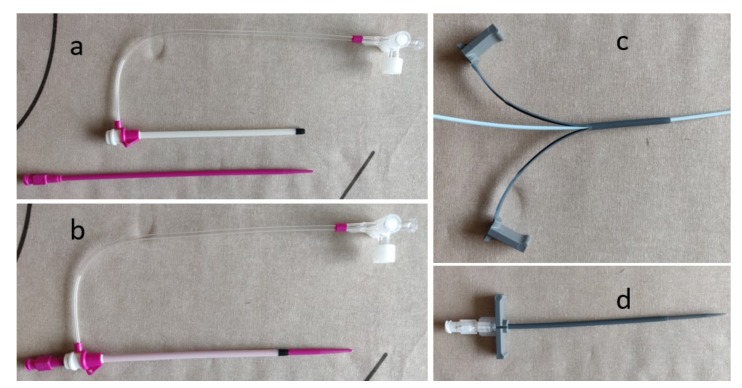
Different access sheaths. (a) Vascular sheath and inner dilator; (b) Assembled vascular sheath with inner dilator; (c) Peel away sheath partially open with inner introducer; (d) Peel away sheath with inner introducer.

Once entry into a vessel is achieved, a sheath is inserted over a wire to maintain access throughout the procedure and prevent access site injury from multiple exchanges (Figure 4). Unlike most other tools used by interventional radiologists, the size of the sheath represents the inner luminal diameter (i.e., an 8F sheath can accommodate an 8F catheter or dilator). Therefore, if a large sheath is required for a procedure and it is determined that serial dilation is needed, dilating to 1–2F sizes larger than the desired sheath is common. This is because the true outer diameter of a sheath is 1.5–2F sizes larger than the inner lumen [3]. Sheaths come in many different diameters, lengths, and shapes. Standard sized sheaths for routine arterial work range from 4F to 8F, although much larger sheaths may be used for aortic interventions or stenting. However, all sheaths have a smooth end in order for the transition between the inner dilator and sheath to be seamless. The inner dilator is integral to the insertion of a sheath, as the sheath itself is relatively flexible and does not have a smooth taper to the diameter of the guidewire. The inner dilator is stiffer, and has a tapered leading edge that helps create a soft tissue tract and provide support while advancing the sheath. The dilator is subsequently removed upon entry to the target structure. Sheaths often have a hemostatic valve on the trailing end. This prevents back bleeding through the sheath when exchanging devices and prevents possible complications such as air embolism [5]. 

Most sheaths also have a clear side port which allows for contrast injection, the connection of a side flush to prevent thrombus formation within the sheath during the procedure, or the infusion of medications (Figure 4a,b). A peel-away sheath has an extravascular end that has two plastic wings, which allow the sheath to be peeled apart and easily removed from the target structure without having to exchange it over the end of a device (Figure 4c,d). A peel-away sheath is useful when inserting any device that has a bulky end or hub that would not allow for the removal of a regular sheath, such as a mediport or tunneled line. Additionally, peel-away sheaths can be used to facilitate the passage of devices through a tight access track or vascular stenosis. Importantly, longer and stiffer sheaths can be used to straighten tortuous vessels, which will facilitate the entry of various catheters and tools to successfully perform a given procedure [6].

### 2.4. Catheters

**Figure 5:**
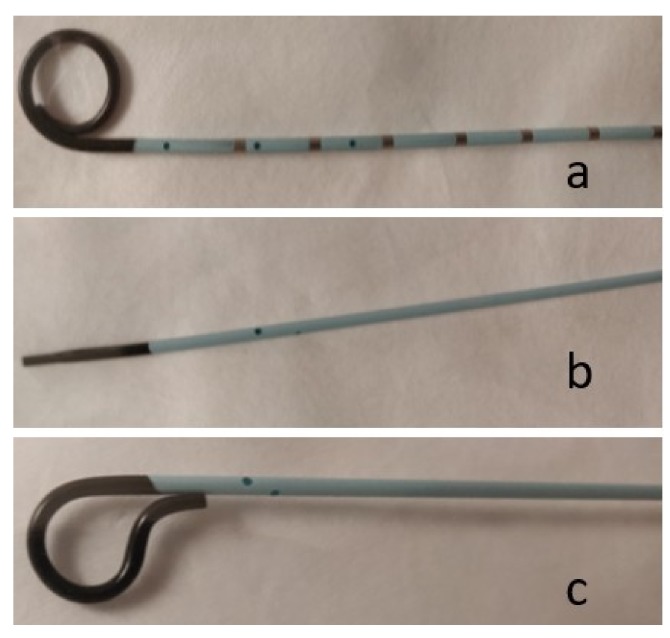
Flush catheters. (a) Flush pigtail catheter, (b) straight flush catheter, and (c) OmniFlush catheter.

**Figure 6:**
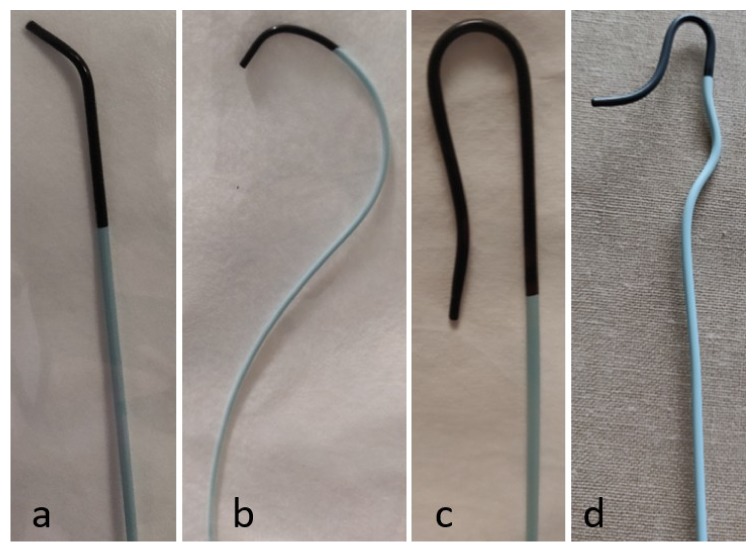
Selective catheters. (a) Berenstein catheter, (b) Cobra 1 cateter, (c) SOS catheter, and (d) Mickelson catheter.

Catheters are used in many different interventional radiology procedures, and therefore come in a variety of shapes, sizes, and configurations. Broadly, catheters can be divided into two main categories: nonselective flush catheters and selective catheters. Flush catheters (Figure 5) allow high-flow injections into large arteries or veins, and most often have multiple side holes to facilitate large volume infusions and prevent vascular injury at the tip of the catheter during power injections [3]. The majority of selective catheters have a single end hole, and therefore require lower flow rates than flush catheters (Figure 6). Whereas flush catheters are designed to have high wall strength, selective catheters are designed for rotational stiffness to accurately transmit manipulation of the trailing end when cannulating a vessel orifice. It is important to consider the shape and length of the tip to ensure that once the vessel is selected, the catheter will not dislodge. In order to decrease the risk of vascular injury, catheters should always be inserted and withdrawn over wires, as the tip of catheters moving within a vessel can cause dissection [7]. The size of the catheter (in French; 3 F = 1-mm diameter) usually represents the outer diameter. The exception is guide catheters, in which the size represents the inner diameter, similar to a sheath.

Important characteristics of catheters include the shape of the leading tip, number and size of side holes, length, pressure rating, volume rating, radiopacity, and material. Selective catheters come in different shapes to accommodate anatomy, particular at vessel origins (Table 2). The choice of selective catheters depends on the size and shape of the targeted vessel. The primary curve of the catheter must approximate the takeoff angle of the targeted vessel to allow for the selection and maintenance of the tip within the ostium of the target vessel. Additionally, certain catheters are designed to ensure the maintenance of the catheter position by opposing the opposite wall of the vessel. For example, the Mikaelsson catheter (Angiodynamics, Queensbury, NY, USA) is often used when catheterizing an aortic branch vessel, as the posterior bulging secondary curve opposes the wall of the aorta and maintains the catheter tip within the small ostium.

Catheter packaging provides information regarding the specific pressure rating of the catheter. This information is important when using the power injector, as exceeding these limits can damage the catheter and the vessel. For selective catheters, side holes are important to reduce the end-hole jet effect. This effect refers to the pressure that pushes back on the catheter when injecting fluid through the tip of the catheter. This pressure can not only dislodge the catheter from the ostium of a vessel, it can also traumatize a vessel [8]. Whereas in flush catheters the side holes are designed to allow for large infusion volumes, in selective catheters, the side holes function to disperse the end-hole jet effects [3]. It is essential to avoid using catheters with side holes for embolization, as it could result in non-target embolization. 

The material of the catheter influences the flexibility, maneuverability, and stability during a given procedure. Similar to wires, catheters can be hydrophilic or non-hydrophilic. Hydrophilic catheters smoothly glide through vessels; however, these catheter have less positional stability and rotational stiffness, and are therefore more difficult to manipulate [9]. Teflon (polytetrafluoroethylene [PTFE]-62 (DuPont, Wilmington, DE, USA)) is a very commonly used catheter material, which is hydrophilic. The material is moderately stiff, and has a high tensile strength and maximal pressure [3]. A commonly used material for selective catheters is polyethylene, as it has little stiffness, which allows it to smoothly follow guidewires into small vessels.

Microcatheters are typically 3F or smaller, and allow for the sub-selection of small vessels, which is critically important in procedures involving the viscera (Figure 5). These microcatheters are advanced through a base or “parent” catheter and used in conjunction with smaller wires (0.014–0.018 inch). Procedures such as chemo/radioembolization and vascular embolization for hemorrhage must be performed very selectively to avoid non-target embolization. The small size of these catheters facilitates precision, whether it be for delivery of chemo/radioembolization in the liver or embolization using coils, glue, or particles for various procedures. When utilizing microcatheters for infusions, saline should be dripped onto the catheter hub before attaching either the power injection or syringe to ensure that no air is present in the line, which could lead to air embolism. There are many microcatheters on the market with different tip shapes (straight, angled, swan-neck) to facilitate the catheterization of vessels. Steerable microcatheters are also available in which the operator can change the shape of the tip through a steering mechanism at the end of the catheter near the hub. 

### 2.5. Organization

Perhaps one of the most critical, yet often overlooked, aspects of performing interventional radiology procedures is organization. For any procedure, whether it be routine such as placing a chemoport or more advanced such as a selective chemo/radioembolization, organization helps minimize the risk of errors. A lack of appropriate planning and equipment unavailability have been shown to account for nearly one-third of interventional radiology errors [1]. While each interventional radiologist will have his or her own preferred organization techniques, there are several important factors to consider when deciding on your own strategy. For example, when using any embolic agent, the table should be clearly divided into two sides with a towel: one side for equipment that will be used with embolics and the other side for non-embolic equipment. Another method is to have a completely separate table for embolic material. These methods ensure that no embolic agent is unintentionally used with resultant non-target embolization (e.g., using a syringe with residual embolic particles to flush a catheter no longer in the target vessel).

**Figure 7:**
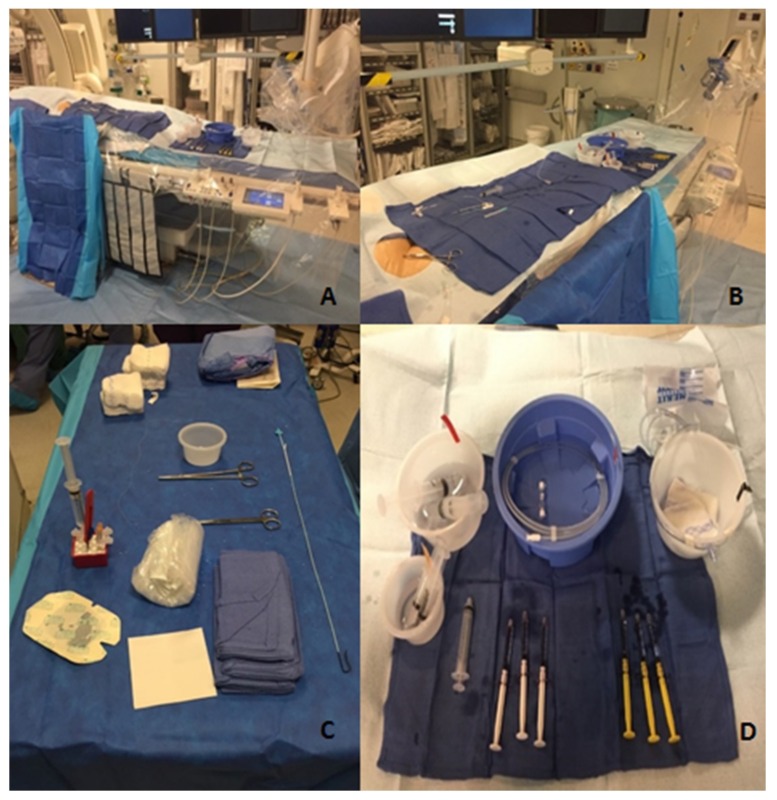
Room organization. (a) Patient table and control; (b) Patient table and power injector; (c) Equipment table; (d) Procedural equipment.

Having a systematic approach to commonly performed procedures ensures a minimization of error, while also increasing the efficiency of the procedure. Figure 7 shows a well-organized room for the treatment of hepatocellular carcinoma with radioembolization via the right femoral artery. These photos were all taken before the procedure began and demonstrate the meticulousness that is needed to ensure organization. The two different colored and labeled syringes are used for saline and contrast, and there is a clear disposal bin for waste. Note how within the disposal bin, there is gauze, which helps prevent any splatter of discarded fluids. The controls for the table are located at the end of the table to allow for room for two operators without having to lean over the controls. The side flush line, as well as the power injector, are set up before beginning the procedure. All the sheaths, dilators, wires, and catheters are flushed and ready for use during the procedure. Additionally, wires are loaded into their appropriate catheters, such as the microwire within the microcatheter. There is ample saline and contrast for the procedure, with commonly used additional supplies ready but not opened so that they are easily available when needed. This thorough set up allows the procedure to proceed smoothly once started, as all needed tools are ready for use, and potentially needed tools are nearby.

The placement of the ultrasound machine in conjunction with the rest of the room set up facilitates successful procedures. While the ultrasound machine is often only needed to gain arterial or venous access at the beginning of a procedure, ensuring operator comfort and maneuverability assists in ease of access. Placing the ultrasound in the direct line of sight of the operator is ideal so that the interventional radiologist can easily see the image without having his or her eyes in one direction while the actual access is in a different direction. Additionally, placing a sterile cover over the ultrasound machine is necessary so that the operator can adjust the settings instead of having an assistant perform this task.

It is important to consider what additional equipment may be required in the setting of a procedural complication. For example, if vessel rupture is a possible complication of a procedure, appropriately sized balloons and/or stents should be on hand in the procedure room, as well as an appropriately sized sheath to deliver the balloon or stent. In general, it is advised to think about what potential complications require immediate intervention and to be as prepared as possible.

### 2.6. Fluoroscopic Imaging Techniques

The most important imaging available for interventional radiologists is fluoroscopy. The basic set up of every fluoroscopy machine has the X-ray source below the patient with the image intensifier/receptor unit above the patient. While this configuration is often known by interventional radiology trainees, the consequences of this set up when maneuvering the C-arm of the fluoroscopy machine is often not fully understood. For example, the use of oblique angles is critically important for determining the anterior versus posterior relationship of structures and procedural equipment (e.g., access needle, catheters) during two-dimensional fluoroscopy. When placing the C-arm in a left anterior oblique (LAO) projection, the image receptor moves toward the left side of the patient. For a supine patient, LAO projection will result in anteriorly located structures moving toward the left side of the display monitor. The opposite is true for right anterior oblique (RAO) projection. Understanding the implications of LAO/RAO projections will allow the operator to assess the depth and the relative anterior–posterior relationship of structures to guide interventions [10]. A common application of LAO projection is when using left radial access to pass a wire down the aorta. When the tip of the wire is at the junction of the left brachiocephalic artery and the aorta, it is difficult to determine whether the wire and catheter are facing toward the ascending or descending aorta. By placing the image receptor in LAO projection, the descending aorta, which is posterior, will be toward the right side of the screen. Therefore, guiding the wire tip toward the right will ensure passage down the descending aorta instead of toward the ascending aorta. Other applications include fluoroscopic guided access during percutaneous transhepatic cholangiography; having different oblique views would assist in determining the position of the access needle in relation to the opacified biliary tree. Another method to determine the location of structures and procedural equipment is to assess the relationship of the structure to the spine, and possibly radiopaque equipment such as a needle driver placed over the skin, while using different obliquities. If the structure and spine move in the same direction while rotating the image receptor, the structure is posterior. 

### 2.7. Imaging and Angiographic Projections 

The two-dimensional nature of fluoroscopy often presents difficulties to trainees when determining the direction of a catheter tip. However, there is a simple technique for quickly determining whether a catheter tip is anterior or posterior, which will assist in catheterizing vessels. From a femoral approach, turning the catheter clockwise (to the left of the patient) will result in the tip moving from the patient’s right to left if it is anterior. Therefore, on the screen in the IR suite, the tip should appear to move to the right-hand side of the screen. In contrast, if the tip moves to the left-hand side of the screen, you know that the tip of the catheter is pointing posteriorly. Importantly, the opposite is true when performing procedures via the radial approach due to the catheter passing over a fulcrum as it ascending through the left arm and then descending through the thoracic aorta. After advancing to the abdominal aorta, turning the catheter clockwise will result in the tip moving from the patient’s left to right, and therefore will move the left-hand side of the screen if the tip is anterior.

### 2.8. Fluoroscopy Image Control and Radiation Exposure

There are several techniques in fluoroscopy to both increase image quality and decrease radiation exposure to both the patient and the operator. While the main source of radiation to the patient is directly from the beam, exposure to the operator mostly occurs from scatter radiation [11]. Overall, collimation, a decreased air gap, higher kVp, pulsed fluoroscopy, and the usage of a large image receptor with collimation reduces radiation exposure [11,12,13]. Collimation restricts the amount of radiation reaching the patient by reducing the cross-sectional area of the X-ray beam, and therefore decreasing the radiation dose delivered to the patient. By reducing the volume of irradiated tissue, the scatter decreases, resulting in a lower dose to the operator and improved image quality (i.e., better image contrast, less image noise). 

The distance from the patient to the image receptor is known as the air gap. By decreasing the air gap, there is less distance for the X-ray beams to scatter after passing through the patient, which reduces the scatter reaching the operator. Fluoroscopy machines also often employ automatic brightness control, which controls the voltage (kVp) and current (mA) of the X-ray beam to ensure that the produced images has a certain clarity and brightness [14]. If the air gap is large, the fluoroscopy machine will automatically increase the radiation of the beam in order to produce a quality image. Therefore, decreasing the air gap will decrease the radiation needed to produce a high-quality image. Additionally, the use of a larger image receptor will help absorb more X-rays and decrease the scattered radiation to the operator.

Although most fluoroscopy machines employ automatic control of the X-ray beam to produce high-quality images, it is important to understand the factors that contribute to the radiation that is produced. The amount of X-rays produced is determined by the current, measured in mA, and the maximum photon energy (voltage), measured in kVp of the X-ray generator. An increase in current directly increases the amount of X-ray photons produced at a given voltage, whereas increasing the voltage by only 15% doubles the amount of X-rays [15]. Therefore, the general rule is to use the highest kVp to achieve the minimally acceptable image quality. 

Pulsed fluoroscopy refers to short bursts of X-ray radiation, instead of constant production. Increasing the pulse rate increases the temporal resolution, but also directly increases the radiation dose [16,17]. Temporal resolution refers to the ability to see real-time image changes. For example, if the pulse rate is set to two frames per second, when manipulating equipment, it may appear to jump from place to place. If the pulse rate is increased, the moving equipment will appear to move more smoothly, as there is decreased time between acquired images. The operator must determine when it is appropriate to increase or decrease the pulse rate based on a need for temporal resolution while minimizing radiation exposure [18]. The dose rate falls in proportion to the pulse rate. However, this relationship is not 1:1; a 50% reduction in the pulse rate results in a 30% reduction of the dose.

Other considerations for radiation exposure and image quality are the use of magnification, digital subtraction angiography, and the roadmap feature. There are two types of magnification: electronic and geometric. Electronic magnification increases the detail resolution of an image (improves spatial resolution), at the expense of increased radiation to the patient [12]. Geometric magnification is performed by lowering the table (brings the patient closer to the X-ray source) and moving the image receptor away from the patient. This increases the patient’s and operator’s radiation dose, and it also increases focal spot blur, resulting in lower spatial resolution. 

Digital subtraction angiography (DSA) is often used to help visualize only contrast-opacified vessels. DSA works by combining a pre-injection image with images taken during the injection of contrast. The computer of the fluoroscope then “subtracts” the pre-contrast images from the contrast image to show only the opacified vessels in detail without surrounding structures [19,20]. Road Map (RM) fluoroscopy is a variant of DSA, where an image at peak opacification is stored on the monitor to provide an outline of the vessels to allow for directed therapy [21]. A “mask” is created in which all the structures present on the image are subtracted so that any contrast injected on subsequent frames is all that appears. While both DSA and RM are very useful when performing intravascular procedures, both result in increased radiation delivered by the fluoroscopy machine. Therefore, it is recommended to limit the use of DSA and RM to reduce patient and operator radiation exposure. The optimal position of the image receptor is achieved by moving the patient away from the X-ray tube (moving table up) and positioning the image receptor as close to the patient as possible. This reduces the patient’s and operator’s dose, and increases image sharpness (better spatial resolution). Regardless of the fluoroscopic procedure being performed, there is always a balance between desired image acquisition and quality, and radiation dose to the patient and personnel. The aim is always to keep radiation exposure to a minimum while generating appropriate imaging to safely perform the procedure. 

## Figures and Tables

**Table 1:** Categories of wires with examples of available wires, properties, and uses.

	Properties				
	Hydrophilicity	Stiffness	Sizes	Tip	Uses
**Access Wire**					
Cope Mandril (Cook Medical, Bloomington, IN)		++	0.018”	Floppy straight	Initial access (vessel, biliary tree, urinary tract)
Bentson (Cook Medical, Bloomington, IN)	−	+	0.035”	Extremely floppy	Can be used as an access wire in vessels
**Maneuver Wire**					
Fathom (Boston Scientific, Marlborough, MA)	+	+	0.014”, 0.015”	Shapeable or pre-shaped	Sub-select small vessels
Glidewire GT (Terumo Medical, Somerset, NJ)	+	+	0.018”	Straight shapeable; pre-shaped single/double angle curves	Sub-select small vessels
Standard Glidewire (Terumo Medical, Somerset, NJ)	+	+	0.035”	Straight or curved	Sub-select larger vessels
Stiff Glidewire (Terumo Medical, Somerset, NJ)	+	++	0.035”	Straight or curved	Maneuver wire, can be used as rail wire when needed
Standard Zipwire (Boston Scientific, Marlborough, MA)	+	+	0.018”–0.035”	Straight, angled, and J-tip	
Stiff Zipwire (Boston Scientific, Marlborough, MA)	+	++	0.018”–0.035”	Straight, angled, and J-tip	
Nitrex (eV3, Plymouth, MN)	−	+	0.014”, 0.018”, 0.025”	Different tip shapes	Very flexible maneuver wire
**Rail Wire**					
Rosen (Cook Medical, Bloomington, IN)	−	+++	0.035”	Stiff, J-tip	Can be helpful in patients with tortuous vasculature or for access through a hostile or obese groin
Amplatz (Boston Scientific, Marlborough, MA)	+	++++	0.035”	Straight floppy tip	Can be helpful in patients with tortuous vasculature or for access through a hostile or obese groin
Roadrunner (Cook Medical, Bloomington, IN)	+	+++	0.035”	Floppy, angled, straight, or double flexible	
v18 (Boston Scientific, Marlborough, MA)	+	+++	0.018”	Straight floppy shapeable tip	
Lunderquist (Cook Medical, Bloomington, IN)		+++++	0.035”	Straight, curved, or double curved	The stiffest guidewire used for interventions requiring manipulation with large devices; can support the advancement of large sheaths and straighten tortuous arterial segmental
Meier (Boston Scientific, Marlborough, MA)	−	+++++	0.035”	Floppy J or C tip	
**Other Wires**					
Glidewire Advantage (Terumo Medical, Somerset, NJ)	+ 25-cm leading segment	++ Trailing end	0.014”, 0.018”, 0.035”		Stiffer nitinol core on the trailing end for better torque transfer, steerability, and device support during exchanges; can be used as combined maneuver and rail wire
Magic Torque Guide Wire (Boston Scientific, Marlborough, MA)	+ leading 10 cm		0.035”		Have metallic markers spaced 1 centimeter along a floppy tip for measuring

Stiffness of wires in this table are in comparison to same size and type (hydrophilic or non-hydrophilic) wires (“= inches, + reflects degree of hydrophilicity and stiffness.

**Table 2:** Commonly used catheters, along with their uses.

Catheter	Common Uses
Pigtail flush	Aortograms and venograms of large veins
Straight flush	Aortograms and venograms of large veins
Curved flush (e.g., Omni Flush)	Aortograms and venograms of large veins Contralateral iliac selection of contralateral iliac
Angled Pigtail (e.g., Grollman)	Pulmonary artery selection and angiography
Simmons 1, 2, and 3	Celiac, superior mesenteric, inferior mesenteric, and renal artery, navigating from left brachiocephalic artery to descending aorta
SOS	Celiac, superior mesenteric, inferior mesenteric, and renal artery or vein runs, navigating from left brachiocephalic artery to descending aorta
Cobra 1, 2, and 3	Adrenal vein sampling, celiac, visceral artery or vein runs
Vertebral	Uterine artery, subclavian, vertebral artery, and vein runs
Mikaelsson	Bronchial, celiac, superior mesenteric, and inferior mesenteric artery or vein runs
Rosch Inferior Mesenteric (RIM)	
Contralateral I and II	Crossing from one iliac artery or vein to the other
Microcatheters	Gastrointestinal bleeding, delivery of microcoils, delivery of chemo/radiation therapy
